# Hippo pathway in endometriosis pathogenesis: from cellular dysregulation to therapeutic opportunities

**DOI:** 10.3389/fphar.2026.1897505

**Published:** 2026-09-08

**Authors:** Gizemnaz Kara, Aylin Yaba

**Affiliations:** 1 Faculty of Medicine, Yeditepe University, Istanbul, Türkiye; 2 Department of Histology and Embryology, Faculty of Medicine, Yeditepe University, Istanbul, Türkiye

**Keywords:** endometriosis, fibrosis, Hippo signalling pathway, non-hormonal therapy, progesterone resistance, YAP1

## Abstract

Endometriosis is a chronic, heterogeneous gynaecological disorder characterised by the presence of endometrial-like tissue outside the uterine cavity and is associated with pain, infertility, and reduced quality of life. Emerging evidence suggests that dysregulation of the Hippo signalling pathway—a central regulator of cell proliferation, survival, mechanotransduction, and tissue homeostasis—may contribute to several pathogenic processes involved in endometriosis. This narrative review synthesises direct evidence from endometriosis studies and clearly identified mechanistic evidence from related disease models. Aberrant Hippo pathway activity and sustained activation of the transcriptional co-activator YAP1 have been linked to enhanced cellular proliferation, invasive behaviour, apoptosis resistance, altered autophagy, ferroptosis resistance, fibrosis, and progesterone resistance. Hippo signalling integrates mechanical, metabolic, inflammatory, and hormonal cues within the endometriotic microenvironment and interacts with other disease-relevant pathways, including the mechanistic target of rapamycin (mTOR), estrogen and progesterone signalling, epigenetic regulation, and immune modulation. Preclinical and experimental findings suggest that components of the Hippo pathway and its regulatory network may represent potential non-hormonal therapeutic targets. However, no Hippo pathway-targeting strategy has been clinically validated for endometriosis, and the physiological roles of YAP/TAZ in tissue homeostasis and regeneration require careful consideration. Further subtype-resolved mechanistic studies, robust preclinical validation, and clinical safety assessment are, therefore, required before these approaches can be translated into practice.

## 1 Endometriosis

Endometriosis is a chronic and debilitating gynaecological disorder characterised by endometrial-like tissue outside the uterine cavity, commonly involving the ovaries, pelvic peritoneum, and other pelvic structures. Based on anatomical location and pathological features, the disease is conventionally classified into three major subtypes: superficial peritoneal endometriosis (SPE), the most prevalent form; ovarian endometrioma (OE), commonly referred to as an endometrioma or “chocolate cyst”; and deep infiltrating endometriosis (DE). These subtypes may represent biologically distinct entities with differences in inflammatory activity, fibrosis, hormonal responsiveness, and invasive behaviour (Nisolle and Donnez, 1997).

Although the precise etiology of endometriosis remains incompletely understood, the disease is widely considered to arise from a complex interplay of genetic susceptibility, environmental influences, hormonal dysregulation, and altered immune responses. Retrograde menstruation remains the most widely accepted initiating mechanism; however, only a subset of women develop endometriosis, suggesting that additional molecular, inflammatory, and microenvironmental factors contribute to lesion establishment and persistence (Habiba et al., 2023). In severe cases, endometriosis may result in infertility (Ozkan et al., 2008). Several risk factors have been associated with endometriosis, including early menarche, shorter menstrual cycle length, low body mass index (BMI), nulliparity, and congenital obstructive Müllerian anomalies (As-Sanie et al., 2025).

Recent evidence further characterises endometriosis as a chronic inflammatory and fibrotic disorder associated with altered immune responses, extracellular matrix remodelling, progesterone resistance, and aberrant tissue repair, potentially reflecting the repeated microinjury and repair processes proposed in the tissue injury and repair (TIAR) theory (Chapron et al., 2019). Given the established role of Hippo-YAP signalling in mechanosensing and fibrotic tissue remodelling, dysregulation of this pathway may contribute to lesion establishment, persistence, and fibrosis (Liu et al., 2015). Direct comparisons among SPE, OE, and DE are currently lacking; therefore, the possibility of greater Hippo pathway activation in highly fibrotic or invasive lesions, particularly DE, should be regarded as a testable hypothesis rather than an established subtype-specific mechanism (Table 1).

**TABLE 1:** Core components of the Hippo signalling pathway and their functions.

Component	Category	Function in the Hippo pathway	Relevance to endometriosis	Key reference
MST1/2 (STK4/STK3)	Ser/Thr kinases	Initiate Hippo kinase cascade	Reduced activity has been associated with increased YAP activation in ectopic lesions	Boggiano et al. (2011), Callus et al. (2006), Harvey et al. (2013)
SAV1	Scaffold protein	Stabilizes MST1/2–LATS complex	Downregulated in ectopic tissue	Callus et al. (2006), Cui et al. (2020)
MOB1A/B	Adaptor proteins	Required for LATS activation	Decreased phosphorylation in ectopic endometrium	Praskova et al. (2008), Cui et al. (2020)
LATS1/2	Tumour suppressor kinases	Phosphorylate YAP/TAZ	Reduced activity may facilitate nuclear YAP accumulation	(Meng et al. (2015), Harvey et al. (2013), Cui et al. (2020)
YAP1	Transcriptional co-activator	Drives TEAD-dependent transcription	Reported to be overexpressed and transcriptionally active in ectopic lesions	Song et al. (2016), Pei et al. (2019)
TAZ (WWTR1)	Transcriptional co-activator	Functional paralog of YAP	Elevated expression in lesions	Song et al. (2016), Boddeti et al. (2025)
TEAD1–4	Transcription factors	Bind YAP/TAZ	Mediate fibrotic and invasive programs	Zhao et al. (2008), Zhu et al. (2017)

Endometriosis presents with a broad spectrum of clinical manifestations, among which pelvic pain is the most frequently reported symptom and affects approximately 80% of patients with endometriosis (Maddern et al., 2020). This pain commonly manifests as dysmenorrhea, dyspareunia, dyschezia, or pain during urination (dysuria). Endometriosis-associated pain is multifactorial and arises from the interaction of central and peripheral pain mechanisms, driven by lesion-specific pathophysiological processes. These include elevated levels of cytokines, growth factors, prostaglandins, and other inflammatory mediators within the lesion microenvironment, as well as neuroangiogenesis, fibrosis, aberrant tissue remodelling, and altered immune cell activity, all of which contribute to lesion persistence and chronic pain development. Studies have demonstrated that endometriotic lesions and the surrounding peritoneal microenvironment differ fundamentally from eutopic endometrium in terms of inflammatory signalling, steroid hormone responsiveness, neurovascular density, and immune cell composition, particularly involving macrophage-related pathways (Table 2). Collectively, these features link lesion biology to pain pathways and lesion persistence, providing mechanistic insight into disease symptoms while also highlighting potential targets for disease-modifying therapies (Saunders and Horne, 2025). Persistent peripheral inflammation may additionally promote central sensitisation, thereby contributing to chronic pelvic pain in endometriosis (Maddern et al., 2020).

**TABLE 2:** Hippo-YAP-dependent cellular processes dysregulated in endometriosis.

Biological process	Hippo/YAP involvement	Pathological consequence	Key reference
Cell proliferation	Nuclear YAP-TEAD activation	Lesion growth	Song et al. (2016), Cui et al. (2020)
Apoptosis	Suppression of pro-apoptotic genes	Lesion survival	Vetvicka et al. (2016), Song et al. (2016)
Autophagy	YAP-mTOR activation	Impaired decidualisation	Pei et al. (2019), Pei et al. (2022)
Ferritinophagy	Reduced USP33–LATS1 activity and impaired NCOA4-mediated ferritinophagy	Reduced ferroptotic sensitivity and lesion survival	Santana-Codina et al. (2021), Li L. et al. (2026)
Ferroptosis	Antioxidant reprogramming	Ferroptosis resistance	Ni and Li (2024), Sun and Chi (2021), Xiang et al. (2023)
Fibrosis	YAP-driven connective tissue growth factor (CTGF)/extracellular matrix (ECM) induction	Tissue stiffening	(Liu et al. (2015), Zhu et al. (2017)
EMT and invasion	YAP-driven transcription	Enhanced invasive behaviour; possible relevance to fibrotic or deep lesions	Konno et al. (2020), Feng et al. (2025)
Angiogenesis	Hypoxia-YAP signalling	Sustained vascularization	Wu et al. (2019), Lin et al. (2017)

Additional clinical manifestations include heavy or irregular menstrual bleeding, such as intermenstrual bleeding, menorrhagia, or prolonged menstrual periods, as well as infertility, often presenting as difficulty in achieving or maintaining a successful pregnancy. Gastrointestinal symptoms are also common and may include bloating (frequently referred to as “endo belly”), nausea, diarrhea, constipation, or abdominal pain (Velho et al., 2023). Systemic symptoms such as persistent fatigue may occur even after adequate rest. Some patients experience painful ovulation or mid-cycle pain, while others report localised tenderness or pruritus in surgical scars following endometriosis-related procedures. Psychological comorbidities, including anxiety and depression, are frequently reported and substantially impair quality of life (Saunders and Horne, 2021). Collectively, these diverse clinical manifestations reflect the complex inflammatory, neuroimmune, and fibrotic nature of endometriosis.

The severity of symptoms varies widely among patients, and a significant proportion of women remain asymptomatic. The diagnosis of endometriosis typically relies on a combination of detailed medical history, physical examination, and imaging modalities. Ultrasonography and magnetic resonance imaging (MRI) are recommended in several clinical guidelines for the detection of OE and DE. However, these modalities have limited sensitivity for SPE and extrapelvic lesions. Emerging evidence suggests that molecular imaging approaches targeting fibrotic components of endometriotic lesions may improve diagnostic sensitivity and disease characterisation. In this context, radiolabeled molecular targets, such as fibroblast activation protein-α (FAP), which is highly expressed in fibrotic tissue, have emerged as promising alternative diagnostic tools. Furthermore, advanced imaging techniques, including positron emission tomography–computed tomography (PET–CT) combined with specific radiotracers, show potential for the detection of fibrotic endometriotic lesions. However, these approaches remain largely investigational and require further clinical validation before routine implementation. Nevertheless, the diagnostic utility of fluorodeoxyglucose (FDG) is limited by physiological uptake in the endometrium and ovaries of premenopausal women, complicating image interpretation (Saunders and Horne, 2025).

In selected cases, diagnostic laparoscopy remains necessary to confirm endometriosis, allowing direct visualisation and surgical excision of ectopic lesions (Ozkan and Arici, 2009). According to the Revised American Society for Reproductive Medicine (rASRM) classification system, endometriosis is staged from I to IV based on lesion location, depth of invasion, extent, morphology, and subtype (Author Anonymous, 1997). However, mounting evidence indicates that the correlation between rASRM stage and pain severity is weak and inconsistent, underscoring that pain intensity does not reliably reflect disease stage (Vercellini et al., 2007).

Despite its high prevalence, endometriosis is frequently misdiagnosed or remains undetected for prolonged periods, largely due to limited disease awareness and symptom overlap with other conditions such as irritable bowel syndrome, pelvic inflammatory disease, and bladder pain syndrome. Consequently, patients often consult multiple specialists and receive symptomatic treatment, resulting in repeated healthcare visits and substantial diagnostic delays (Melgaard et al., 2025) (Table 3). Current management strategies remain largely focused on symptom control and suppression of lesion growth; however, these approaches do not adequately address the underlying molecular mechanisms associated with lesion persistence, chronic inflammation, fibrosis and disease recurrence (Saunders and Horne, 2021; Kalaitzopoulos et al., 2021; Bora and Yaba, 2021). This limitation has driven increasing interest in molecular pathways involved in endometriosis pathogenesis, including pathways associated with inflammation, mechanotransduction, fibrosis and aberrant cellular survival signalling. Based on evidence derived from both endometriosis-specific studies and broader mechanobiology research, the Hippo pathway has emerged as a potential regulator linking these processes.

**TABLE 3:** Hippo-YAP signalling and hormonal resistance in endometriosis.

Hormonal pathway	Interaction with YAP	Clinical implication	Key reference
Progesterone receptor (PGR)	YAP induces miR-21-5p	Progesterone resistance	Patel et al. (2017), Lin et al. (2023)
Decidualisation	YAP suppresses autophagy	Implantation failure	Pei et al. (2019), Lin et al. (2023)
Estrogen receptor β (ESR2)	YAP–ESR2 crosstalk	Enhanced invasiveness	Zeng et al. (2020)
Progestin therapy	*In vitro* YAP inhibition increased progestin responsiveness	Potential restoration of sensitivity; not clinically validated	Lin et al. (2023)

Conventional therapeutic approaches primarily include hormonal suppression, analgesics, and surgical excision of lesions (Huntington and Gilmour, 2005). Non-steroidal anti-inflammatory drugs are commonly used for pain management, although the supporting evidence is limited (Brown et al., 2017). Hormonal therapies suppress disease activity but may be limited by adverse effects, contraindications, and symptom recurrence after discontinuation (Petraglia et al., 2025). Laparoscopic excision may reduce pain and improve fertility outcomes in selected patients, although recurrence remains common (Vercellini et al., 2009).

In addition to conventional therapies, lifestyle and complementary interventions have been investigated as supportive approaches to symptom management. Dietary modification, physical activity, and stress-reduction strategies may improve pain perception or quality of life in some patients, although the evidence is heterogeneous (Nirgianakis et al., 2022). A systematic review has examined physical exercise in endometriosis (Bonocher et al., 2014), while more recent work has evaluated broader self-management strategies (Mardon et al., 2023). Acupuncture has also been assessed in a systematic review and meta-analysis (Giese et al., 2023). Natural bioactive compounds and herbal medicines are under experimental investigation for anti-inflammatory, antifibrotic, and signalling-modulatory effects (Elbanna et al., 2025). These findings remain preliminary and do not establish clinical efficacy or Hippo pathway specificity.

Although substantial progress has been made in defining the inflammatory, hormonal, and fibrotic biology of endometriosis, important mechanistic and therapeutic gaps remain. This review, therefore, examines evidence linking Hippo pathway dysregulation to lesion establishment, persistence, and clinical phenotypes while explicitly distinguishing direct endometriosis data from mechanistic evidence extrapolated from related models. It also evaluates experimental therapeutic strategies and the limitations that must be addressed before clinical translation.

## 2 Literature search strategy

PubMed was the sole bibliographic database used for this narrative review. The search covered database inception to 31 July 2026, with no publication-year restriction. English-language, peer-reviewed original studies, translational or clinical investigations, and selected reviews with accessible abstracts and full texts relevant to the review scope were considered.

The principal search strategy combined the disease term with pathway and process terms as follows: (“endometriosis” [Title/Abstract]) AND (“Hippo signalling” [Title/Abstract] OR “Hippo pathway” [Title/Abstract] OR YAP [Title/Abstract] OR TAZ [Title/Abstract] OR mechanotransduction [Title/Abstract] OR fibrosis [Title/Abstract] OR autophagy [Title/Abstract] OR ferroptosis [Title/Abstract] OR “progesterone resistance” [Title/Abstract] OR SOX18 [Title/Abstract] OR OTUB1 [Title/Abstract] OR USP33 [Title/Abstract] OR circRNA [Title/Abstract] OR m6A [Title/Abstract]). Additional targeted combinations were used for immune regulation, lesion subtype, and experimental therapeutic strategies (Table 4).

**TABLE 4:** Upstream regulators of Hippo–YAP signalling in endometriosis.

Regulator	Mechanism	Effect on YAP	Functional outcome	Key reference
ECM stiffness	Actin remodelling	YAP activation	Fibrosis-driven persistence	Aragona et al. (2013), Liu et al. (2015)
Hypoxia	LATS1 suppression	Nuclear YAP accumulation	Angiogenesis, inflammation	Wu et al. (2019), Lin et al. (2017)
SOX18	OTUB1 induction	YAP stabilization	Invasion and fibrosis	Feng et al. (2025)
OTUB1	Deubiquitination	Prevents YAP degradation	Sustained YAP signalling	Feng et al. (2025), Ling et al. (2022)
USP33	LATS1 stabilization through reduced ubiquitination	Indirect YAP inhibition	Restored ferritinophagy and ferroptotic sensitivity in one endometriosis study	Li J. et al. (2026), Li L. et al. (2026)
ANTXR2	Promotes nuclear YAP	Transcriptional activation	Lesion progression	Bell et al. (2001), Scobie et al. (2003), Liu et al. (2009), Lin et al. (2019)
circRNAs	miRNA sponging	YAP derepression	EMT induction	Zhang et al. (2018), Wang et al. (2020)
m6A modification	Reduced METTL3/METTL14 expression and global m6A hypomethylation	Association with Hippo-related transcript networks; direct causality unconfirmed	Enhanced proliferation and invasion after METTL3/METTL14 co-silencing	Wu et al. (2023), Li et al. (2019), Li et al. (2022), Shen et al. (2023)

Titles and abstracts were screened for relevance, followed by full-text evaluation. Studies directly investigating Hippo signalling in endometriosis were prioritised. Mechanistically relevant studies from other gynaecological, oncological, or fibrotic models were included only when direct endometriosis evidence was unavailable and are identified as indirect evidence in the text. Conference abstracts, editorials, duplicate reports, non-peer-reviewed material, and studies without a relevant Hippo-endometriosis or mechanistic link were excluded. Reference lists of eligible articles were also screened manually.

Because the work was designed as a narrative review, duplicate removal and PRISMA-style numerical tracking were not prospectively recorded, and no formal risk-of-bias assessment was performed. The final synthesis contains 94 cited publications. This limitation is stated explicitly to avoid presenting the review as a systematic review.

## 3 Hippo signalling pathway

The Hippo signalling pathway, also referred to as the Salvador–Warts–Hippo (SWH) pathway, is an evolutionarily conserved regulatory network that governs tissue homeostasis by controlling cell proliferation, apoptosis, and organ size. Initially characterised in *Drosophila melanogaster*, the pathway was shown to restrict tissue overgrowth by coordinating cell-cycle progression and programmed cell death. Beyond its classical role in growth control, the Hippo signalling functions as a mechanosensitive network that integrates biochemical, inflammatory, and biomechanical cues from the cellular microenvironment.

In mammals, the core Hippo pathway consists of mammalian STE20-like kinases 1 and 2 (MST1/2), the scaffold protein Salvador homolog 1 (SAV1), MOB kinase activator 1A/B (MOB1A/B), large tumour suppressor kinases 1 and 2 (LATS1/2), the transcriptional co-activator Yes-associated protein 1 (YAP), and the transcriptional co-activator with PDZ-binding motif (TAZ; also known as WWTR1). In the canonical Hippo pathway, activation of MST1/2 and LATS1/2 results in phosphorylation-dependent inhibition of YAP and TAZ, promoting their cytoplasmic retention and degradation. Conversely, suppression or dysregulation of Hippo kinase activity permits nuclear accumulation of YAP/TAZ, where they interact primarily with TEAD transcription factors to regulate genes involved in proliferation, survival, fibrosis, mechanotransduction, cellular plasticity and tissue remodelling.

The Hippo kinase cascade is initiated by thousand-and-one amino acid (TAO) kinases, a family of serine/threonine protein kinases comprising TAO1, TAO2, and TAO3, which phosphorylate and activate MST1/2 (Boggiano et al., 2011). Activated MST1/2, in complex with SAV1, subsequently recruit and phosphorylate LATS1/2 in association with MOB1A/B (Callus et al., 2006; Praskova et al., 2008). Activated LATS1/2 phosphorylate YAP and TAZ, promoting their cytoplasmic retention and functional inhibition. In parallel, members of the MAP4K family (MAP4K1/2/3/5) can activate LATS1/2 independently of MST1/2, providing regulatory redundancy (Meng et al., 2015). Collectively, these kinase networks constrain YAP/TAZ activity and preserve tissue homeostasis under physiological conditions.

Additional upstream regulatory mechanisms further modulate Hippo pathway activity. Among these, the striatin-interacting phosphatase and kinase (STRIPAK) complex negatively regulates both MST1/2 and MAP4Ks, thereby suppressing Hippo signalling (Seo et al., 2020). Simultaneous depletion of MST1/2 and MAP4Ks results in profound impairment of downstream Hippo pathway activity, highlighting the importance of coordinated upstream regulation.

Beyond canonical kinase-dependent regulation, YAP/TAZ activity responds to cell density, substrate stiffness, cellular tension, extracellular matrix (ECM) composition, soluble factors, and G protein-coupled receptor signalling (Gaspar and Tapon, 2014). YAP accumulates in the nucleus of sparsely populated cells but is sequestered in the cytoplasm at high cell density (Zhao et al., 2007). Increased ECM rigidity and mechanical stress enhance YAP/TAZ activity through actin-cytoskeleton remodelling, particularly F-actin assembly (Aragona et al., 2013). Mechanical inputs can also regulate YAP/TAZ independently of the core Hippo kinases. Cellular stressors, including hypoxia, osmotic imbalance, heat shock, and energy deprivation, further influence Hippo signalling (Fu et al., 2022). In endometriosis, chronic inflammation, fibrosis, hypoxia, and ECM remodelling may, therefore, create a permissive environment for aberrant YAP/TAZ activation. During menstruation, these mechanosensitive responses may intersect with hypoxia-inducible factor 1-alpha (HIF-1α)/YAP signalling and influence endometrial repair or ectopic lesion establishment (Zhang et al., 2022).

Given its central role in cell fate and tissue growth, Hippo pathway dysregulation has been extensively studied in cancer and fibrotic diseases. Aberrant Hippo signalling can enhance proliferation, disrupt polarity, suppress apoptosis, and sustain YAP/TAZ–TEAD-dependent survival programmes (Yeung et al., 2019). Persistent YAP/TAZ activation has also been associated with fibrotic progression in the liver, kidney, lung, heart, and female reproductive tissues. In experimental fibrotic models, sustained YAP activation is linked to increased tissue stiffness and progressive fibrosis (Liu et al., 2015). YAP/TAZ activity has additionally been associated with treatment resistance and enhanced survival in several cancer models (Bartucci et al., 2015). These findings provide mechanistic context but should not be considered direct evidence in endometriosis.

Although mutations in core Hippo pathway components are relatively uncommon in human cancers, functional pathway dysregulation is frequent. Mouse and *Drosophila* models show that loss of MST1/2 or LATS1/2, or constitutive YAP/TAZ activation, can drive tissue overgrowth and tumour formation. In human malignancies, altered upstream regulation—rather than direct mutation of core components—often underlies Hippo dysfunction (Harvey et al., 2013). Sustained YAP/TAZ activity can promote stem-like properties, metastatic potential, and chemoresistance (Zanconato et al., 2016). These observations support investigation of the pathway in other proliferative and fibrotic disorders while also underscoring the need to separate cross-disease mechanistic inference from endometriosis-specific evidence.

## 4 Hippo signalling dysregulation in endometriosis

**FIGURE 1:**
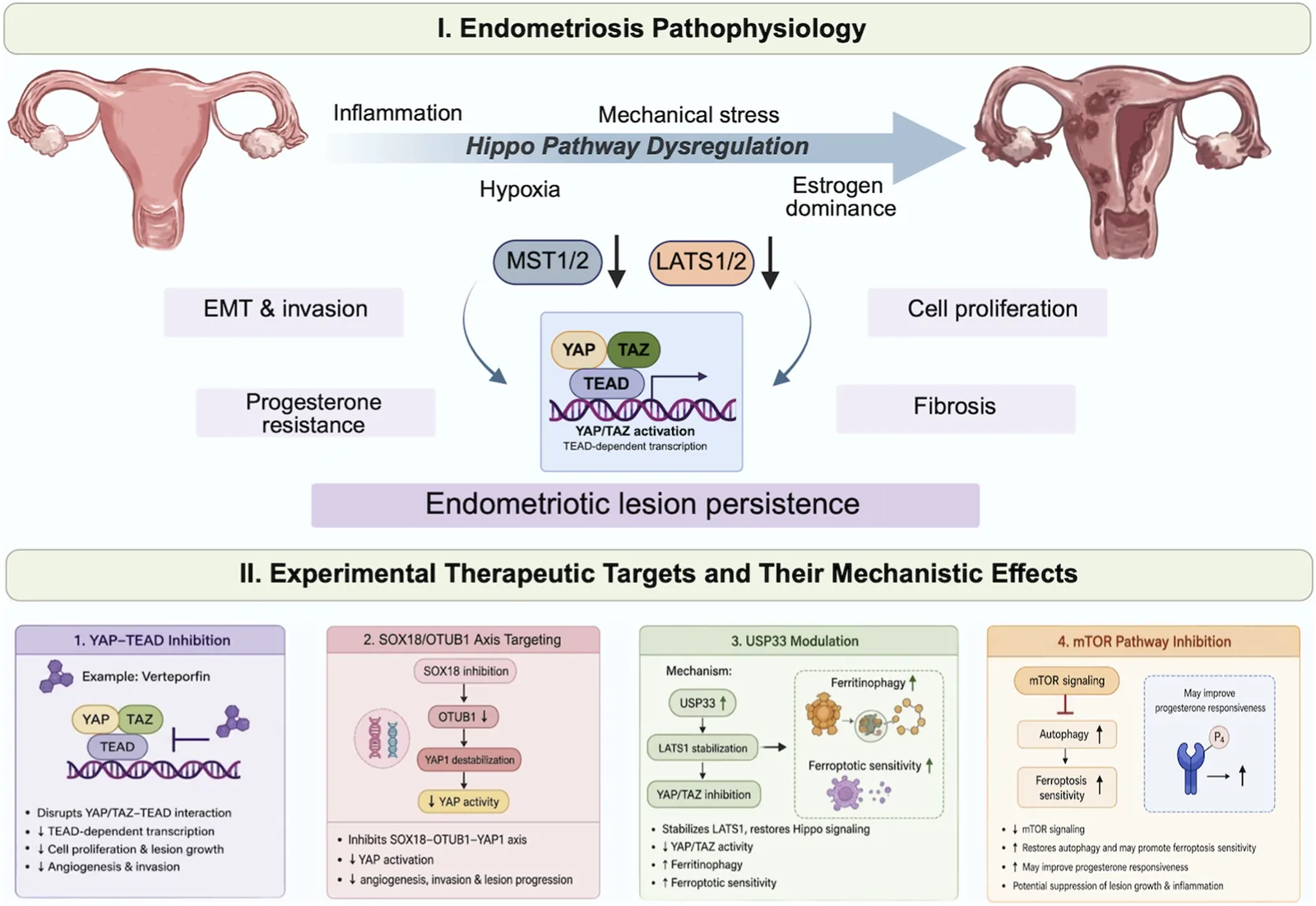
Proposed model of Hippo pathway dysregulation in endometriosis. Mechanical and microenvironmental cues, including extracellular matrix (ECM) stiffness, hypoxia, reactive oxygen species (ROS)/hypoxia-inducible factor 1-alpha (HIF-1α) signalling, inflammation, ANTXR2, the SOX18–OTUB1 axis, circular RNAs, and altered epitranscriptomic regulation, converge on the MST1/2–SAV1–MOB1–LATS1/2 kinase cascade. Reduced LATS activity permits nuclear YAP/TAZ–TEAD signalling and may promote proliferation, survival, fibrosis, epithelial–mesenchymal transition, angiogenesis, autophagy impairment, ferroptosis resistance, and progesterone resistance. Solid arrows denote direct endometriosis evidence; dashed arrows denote mechanisms inferred from related disease models. ANTXR2, anthrax toxin receptor 2; OTUB1, OTU domain-containing ubiquitin aldehyde-binding protein 1; SOX18, SRY-box transcription factor 18; TAZ, transcriptional co-activator with PDZ-binding motif; YAP1, yes-associated protein 1. Created in BioRender. Kara (2026) https://BioRender.com/86o2i2m.

Aberrant regulation of cell proliferation, survival, apoptosis, and tissue remodelling is a defining feature of endometriosis, although the responsible molecular networks remain incompletely understood (Vetvicka et al., 2016). The Hippo pathway is a central regulator of tissue homeostasis, but its contribution to endometriosis has only recently been investigated (Ehmer and Sage, 2016). Available studies implicate altered Hippo-YAP signalling in lesion establishment, persistence, and progression and identify the pathway as a candidate experimental target rather than an established therapy (Song et al., 2016; Boddeti et al., 2025). Unless a subtype is explicitly stated, the cited tissue studies analysed ectopic endometriotic tissue without distinguishing SPE, OE, and DE. Figure 1 summarises direct endometriosis evidence and mechanisms inferred from related models using distinct graphical conventions.

Comparative analyses of ectopic endometrium (EC), eutopic endometrium (EU), and endometrium from women without endometriosis (NE) have demonstrated alterations in canonical Hippo pathway components. Cui et al. (2020) reported increased YAP and TAZ protein expression in EC, accompanied by reduced MOB1, phosphorylated MOB1, SAV1, LATS1, and LATS2. These findings are consistent with suppression of canonical Hippo signalling and increased YAP/TAZ activity in ectopic lesions (Table 5). However, the study did not provide a direct comparison among SPE, OE and DE, and the results should not be interpreted as subtype-specific.

**TABLE 5:** Therapeutic strategies targeting Hippo signalling in endometriosis.

Strategy	Target	Mechanism	Therapeutic potential	Key reference
Verteporfin	YAP–TEAD	Transcriptional blockade	Preclinical candidate; non-selective and not clinically validated for endometriosis	Sun and Chi (2021), Lin et al. (2023)
SOX18 inhibition	SOX18–OTUB1	Reduced YAP stability	Hypothesis-generating antifibrotic strategy requiring independent validation	Feng et al. (2025)
OTUB1 targeting	Deubiquitinase	Promotes YAP degradation	Hypothesis-generating strategy to reduce YAP-dependent lesion survival	Feng et al. (2025)
USP33 activation	Ferritinophagy	Restores ferroptosis	Single-study preclinical strategy to restore ferroptotic sensitivity	Li L. et al. (2026)
mTOR inhibitors	YAP downstream	Autophagy restoration	Preclinical strategy requiring evaluation of systemic mTOR effects	Pei et al. (2019), Pei et al. (2022)
Combination therapy	YAP + progestins	Overcomes resistance	Experimental combination strategy; clinical benefit unproven	Lin et al. (2023)
Hu-Po-San	Multi-pathway network modulation	Hippo, inflammatory and fibrotic pathway modulation	Single-study preclinical formulation with uncertain active constituents and target specificity	Huo et al. (2025)

Hippo-YAP signalling may also participate in physiological endometrial repair during menstruation. Mechanical changes in ECM stiffness, together with reactive oxygen species (ROS)/hypoxia-inducible factor 1-alpha (HIF-1α) signalling, regulate YAP activity during endometrial regeneration (Zhang et al., 2022). In endometriosis, persistent inflammatory and hypoxic conditions may dysregulate these repair-associated responses and enhance ectopic cell survival. Endometriosis-specific experimental studies support a role for hypoxia-mediated YAP1 nuclear translocation in lesion progression (Lin et al., 2017). Within the TIAR framework, repeated tissue breakdown and repair may therefore create a permissive environment for aberrant YAP/TAZ activation after retrograde menstruation (Habiba et al., 2023). Findings in adenomyosis further suggest that suppression of canonical Hippo signalling can accompany epithelial–mesenchymal transition (EMT), proliferation and apoptosis resistance, but this represents supportive evidence from a related gynaecological disorder rather than direct evidence in endometriosis (Jin et al., 2023).

Endometriosis is increasingly recognised as a disorder characterised by disrupted crosstalk between autophagy and apoptosis, which may support ectopic cell survival under adverse microenvironmental conditions (Kobayashi et al., 2024). The Hippo–YAP axis can regulate autophagy through the mechanistic target of rapamycin (mTOR). *In vitro* studies showed that YAP overexpression reduced autophagic flux, decreased the LC3-II/LC3-I ratio, and increased mTOR expression (Pei et al., 2019). YAP, TEAD1, and mTOR transcripts were also elevated in eutopic endometrial stromal cells (ESCs) from women with endometriosis. Rapamycin promoted decidualisation *in vitro*, whereas verteporfin did not reproduce this effect, indicating that the YAP–mTOR relationship and decidualisation phenotype require cautious interpretation (Pei et al., 2019). Transcriptomic analyses identified YAP-centred interaction networks associated with autophagy regulation in eutopic ESCs (Pei et al., 2022). Collectively, these findings support a possible YAP–mTOR–autophagy link, but the evidence remains predominantly cell-based.

Beyond global autophagy, selective autophagic processes such as ferritinophagy may link Hippo signalling to iron-dependent cell death (Pei et al., 2022). Ferritinophagy is mediated primarily by nuclear receptor coactivator 4 (NCOA4) and contributes to iron homeostasis and ferroptosis, an iron-dependent form of regulated cell death driven by lipid peroxidation (Santana-Codina et al., 2021; Dixon et al., 2012). Despite recurrent haemorrhage and iron accumulation, endometriotic lesions can display reduced ferroptotic sensitivity (Ni and Li, 2024). The broader proposal that YAP/TAZ reprogramme antioxidant defences and limit lipid peroxidation is supported mainly by cancer and other disease models (Sun and Chi, 2021). Likewise, the E-cadherin–NF2–LATS–YAP/TAZ axis has been linked to density-dependent ferroptosis in non-endometriosis systems (Xiang et al., 2023). These mechanisms provide a biologically plausible framework but should not be presented as fully established in endometriosis.

Direct endometriosis evidence has recently implicated ubiquitin-specific peptidase 33 (USP33) in the regulation of ferritinophagy and ferroptosis. Patient-derived tissue and mechanistic experiments showed that reduced USP33 was associated with increased LATS1 ubiquitination, enhanced YAP activity, impaired ferritinophagy and reduced ferroptotic cell death. Restoration of USP33 stabilised LATS1, reactivated Hippo signalling, and increased ferroptotic sensitivity (Li J. et al., 2026). This study identifies a potential USP33–LATS1–YAP axis in endometriosis; however, it currently represents a single recent report and requires independent replication. Broader conclusions regarding YAP/TAZ-mediated ferroptosis should therefore remain explicitly distinguished from this direct but still limited endometriosis evidence.

Additional regulators of YAP activity contribute to endometriosis-associated invasive phenotypes. Apoptosis-stimulating protein of p53-2 (ASPP2), a tumour suppressor involved in p53-dependent apoptosis and epithelial polarity via PAR3 interaction (Sottocornola et al., 2010; Vives et al., 2006), has been implicated in YAP-dependent cell migration and invasion. Experimental suppression of ASPP2 reduces lipolysis-stimulated lipoprotein receptor (LSR) expression and induces YAP1 activation, promoting migratory and invasive behaviour in endometrial cells. Conversely, YAP1 knockdown restores ASPP2 function, indicating that ASPP2-mediated effects on cell motility are YAP-dependent (Konno et al., 2020).

SRY-box transcription factor 18 (SOX18) has emerged as an upstream regulator of YAP signalling. SOX18 has physiological roles in development and tissue homeostasis (Saitoh and Katoh, 2002; Downes and Koopman, 2001), whereas pathological overexpression can promote proliferation, migration, invasion, and ECM remodelling in other disease contexts (Chen et al., 2024; Chen et al., 2020; Yin et al., 2017). Patient-derived lesion analyses and functional experiments reported increased SOX18 expression in ectopic endometriotic tissues and showed that SOX18 increased OTU domain-containing ubiquitin aldehyde-binding protein 1 (OTUB1), thereby stabilising YAP1 and promoting invasive and fibrotic programmes (Feng et al., 2025). Stabilised YAP1 can interact with TEAD transcription factors and induce fibrotic and invasive gene programmes (Zhao et al., 2008; Zhu et al., 2017). The proposed SOX18–OTUB1–YAP1 circuit is therefore mechanistically compelling, but its reproducibility, subtype distribution and clinical relevance remain to be established.

Hormonal dysregulation remains central to endometriosis pathophysiology, particularly estrogen dominance and progesterone resistance (Patel et al., 2017; Marquardt et al., 2019). YAP1 has been implicated in progesterone resistance through upregulation of microRNA-21-5p (miR-21-5p) and repression of progesterone receptor (PGR) expression. *In vitro* pharmacological experiments showed that verteporfin reduced miR-21-5p and increased progestin responsiveness, while dienogest also reduced YAP1 and miR-21-5p expression (Lin et al., 2023). These cell-based findings support a possible YAP1–miR-21-5p–PGR mechanism but do not yet establish clinical benefit from YAP1 inhibition.

Complex interactions between YAP1 and estrogen signalling have also been reported. Patient-derived cell-based experimental studies in ovarian endometriosis have demonstrated that YAP1 negatively regulates estrogen receptor β (ESR2), thereby modulating the invasive behaviour of endometriotic stromal cells. Knockdown of YAP1 increases ESR2 expression, while combined suppression of YAP1 and ESR2 markedly reduces cellular invasion, indicating that YAP1 influences lesion invasiveness in part through ESR2-dependent mechanisms (Zeng et al., 2020).

Adaptation to hypoxia is a hallmark of ectopic lesion survival. Hypoxic conditions can reduce LATS1 expression, activate YAP1 and induce programmes associated with angiogenesis, steroidogenesis, inflammation and proliferation. Experimental disruption of hypoxia-induced YAP1 signalling using small interfering RNA (siRNA) or pharmacological inhibitors reduced lesion-related phenotypes in preclinical models (Wu et al., 2019; Lin et al., 2017). These findings support a role for YAP1 in hypoxia-associated progression but do not establish a clinically validated intervention.

Emerging evidence suggests that Hippo signalling may also influence the immune microenvironment of endometriosis. A recent study reported that endometriosis-derived exosomes contained reduced miR-196a-5p and that experimental restoration of exosomal miR-196a-5p activated Hippo signalling in macrophages, increased M1-associated markers, and suppressed M2 polarisation (Lu et al., 2025). Pharmacological inhibition of Hippo signalling attenuated these effects, supporting a miR-196a-5p–Hippo-dependent mechanism. Thus, loss of exosomal miR-196a-5p may contribute to a pro-lesional macrophage phenotype, whereas restoration appears protective in the reported experimental system. This conclusion is currently based on a single study and requires validation in independent cohorts, additional immune-cell populations and *in vivo* models.

Post-transcriptional regulation further expands the Hippo network in endometriosis. Circular RNAs (circRNAs) can act as microRNA (miRNA) sponges and show tissue-specific expression patterns (Memczak et al., 2013; Conn et al., 2015). A transcriptomic study of ovarian endometriosis identified circ_101,102 and circ_103,470 among circRNAs associated with Hippo and mTOR-related networks and EMT-related bioinformatic predictions (Zhang et al., 2018). These associations were primarily transcriptomic and were not independently replicated. A separate patient-tissue and *in vitro* study reported that circular RNA ATRNL1 (circATRNL1) promoted EMT through YAP1 upregulation (Wang et al., 2020). Accordingly, circRNA-mediated Hippo regulation is supported by a small number of studies with different designs, and independent functional validation remains necessary.

Anthrax toxin receptor 2 (ANTXR2), a transmembrane protein initially characterised in vascular biology and anthrax toxin signalling (Bell et al., 2001; Scobie et al., 2003; Liu et al., 2009), has been implicated in Hippo–YAP regulation in endometriosis (Lin et al., 2019). Patient-derived tissue analyses and experimental studies reported ANTXR2 overexpressed in endometriotic lesions and an association with increased nuclear YAP1. Hypoxia-mediated reduction of enhancer of zeste homolog 2 (EZH2) was proposed to increase ANTXR2 expression, linking epigenetic regulation, hypoxia, and Hippo signalling (Lin et al., 2019). These findings identify a potential ANTXR2–YAP axis, but replication and subtype-specific validation are still required.

Epitranscriptomic regulation through N6-methyladenosine (m6A) may also contribute to endometriosis-associated signalling networks. The m6A writer complex includes methyltransferase-like 3 (METTL3) and methyltransferase-like 14 (METTL14), which regulate RNA processing, stability, and translation (Fu et al., 2014; Wang et al., 2016; Huang and Chen, 2023). Studies in reproductive biology and related disease models show that altered m6A regulation can affect proliferation, invasion, and EMT (Wu et al., 2023; Li et al., 2019; Li et al., 2022). In endometriosis, Shen et al. (2023) reported reduced METTL3 and METTL14 expression and global m6A hypomethylation in ectopic endometrium; experimental co-silencing of METTL3 and METTL14 increased proliferative and invasive phenotypes. Pathway analysis linked hypomethylated transcripts to several signalling networks, including Hippo signalling, but a direct causal METTL3/METTL14–Hippo–YAP mechanism was not demonstrated. The evidence should therefore be considered preliminary and hypothesis-generating.

## 5 Experimental therapeutic opportunities and future directions

**FIGURE 2:**
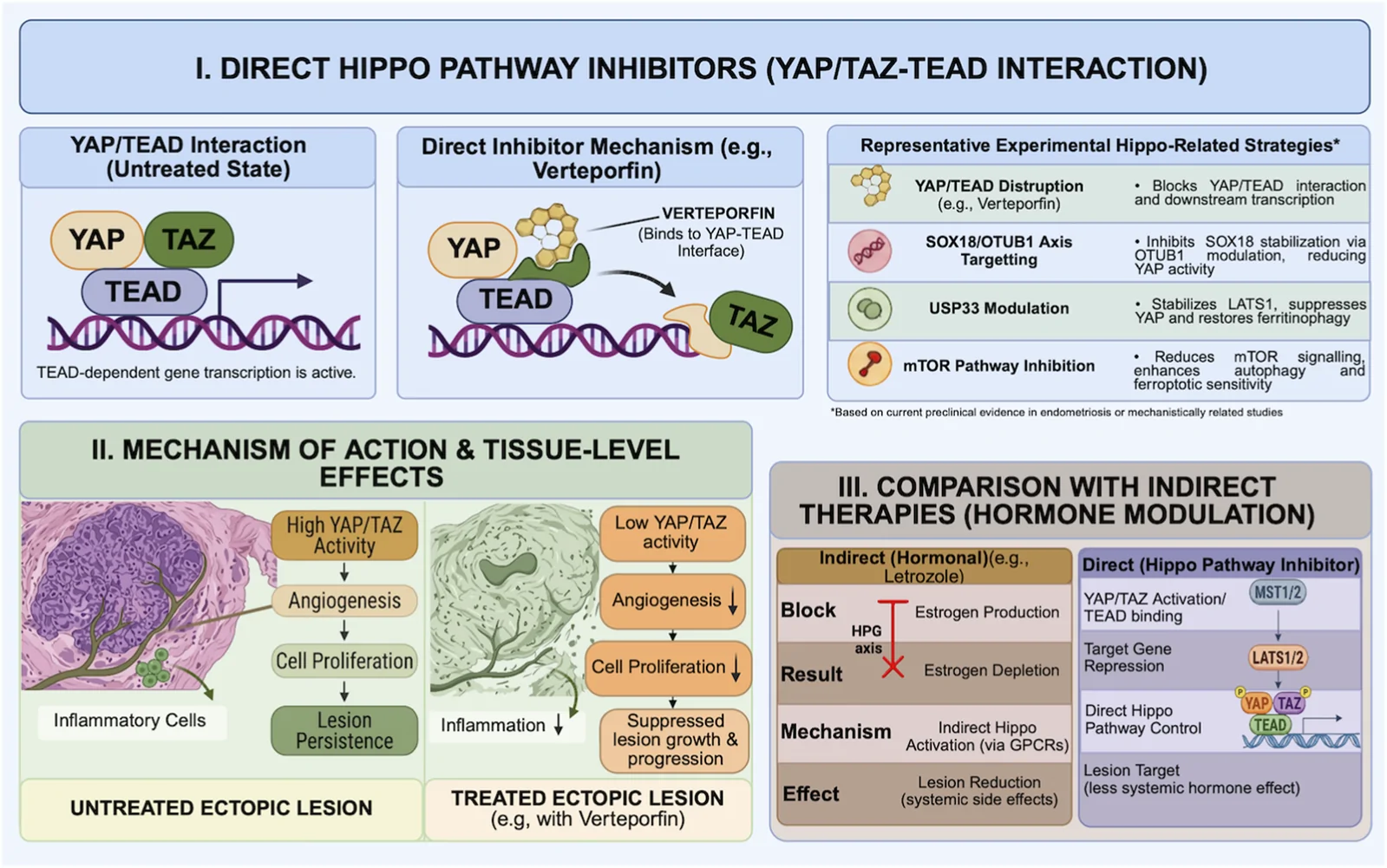
Experimental intervention points within the Hippo regulatory network in endometriosis. Proposed strategies include disruption of YAP–TEAD transcription with verteporfin or related experimental compounds; inhibition of SOX18 or OTUB1 to reduce YAP1 stabilisation; restoration of USP33–LATS1 signalling to increase ferritinophagy and ferroptotic sensitivity; modulation of the YAP–mTOR–autophagy axis; and experimental combination with progestins. Hu-Po-San is shown as a multi-component preclinical formulation with network-level effects rather than a single-target drug. None of these strategies has been clinically validated for endometriosis, and verteporfin is not a selective Hippo pathway inhibitor. mTOR, mechanistic target of rapamycin; USP33, ubiquitin-specific peptidase 33. Created in BioRender. Kara. (2026) https://BioRender.com/dpveide.

Before Hippo pathway-directed strategies can be considered for clinical testing, their efficacy, safety, tissue specificity, and disease subtype dependence require robust preclinical evaluation. Aberrant YAP1 activity provides a rationale for translational research (Song et al., 2016; Cui et al., 2020), but currently available inhibitors are neither endometriosis-specific nor clinically validated. Verteporfin and related experimental YAP–TEAD-disrupting approaches should first be assessed in well-characterised cell, organoid and animal models for lesion burden, pain-related outcomes, recurrence, and systemic toxicity (Sun and Chi, 2021; Xiang et al., 2023). Combination approaches, including experimental YAP modulation with progestins, may be particularly relevant to progesterone-resistant phenotypes but remain supported mainly by cell-based evidence (Patel et al., 2017; Marquardt et al., 2019; Lin et al., 2023). Figure 2 maps the proposed intervention points and explicitly identifies their preclinical status.

Experimental strategies may also target upstream regulators that stabilise or activate YAP1. SOX18 or OTUB1 inhibition could, in principle, destabilise YAP1, but these approaches remain hypothesis-generating and require independent validation (Feng et al., 2025). Restoration of USP33 may increase ferritinophagy and ferroptotic sensitivity through the LATS1–YAP axis; however, this proposal currently rests on a single endometriosis study (Li L. et al., 2026). The YAP–mTOR–autophagy relationship likewise provides a possible metabolic intervention point, although pathway complexity and the physiological functions of mTOR and YAP necessitate careful dose, timing, and tissue-specificity studies (Pei et al., 2019; Pei et al., 2022).

Hu-Po-San (HPS) is a multi-component traditional Chinese herbal preparation composed of several herbal medicines rather than a single defined pharmacological compound; its precise formulation is study-specific and should be interpreted accordingly. In a rat model of endometriosis, multi-omics, network pharmacology and *in vivo* validation associated HPS treatment with activation of canonical Hippo signalling, reduced YAP-associated activity, reduced proliferation and angiogenesis, modulation of inflammatory pathways and increased apoptosis (Huo et al., 2025). The study reported reduced ectopic lesion growth and no detectable toxicity within the evaluated endpoints. Nevertheless, these findings derive from a single preclinical study; the active constituents and target specificity remain uncertain, and independent replication and human safety data are required.

Several mechanistic priorities remain. Human studies should validate the SOX18–OTUB1–YAP1 axis across disease stages and lesion subtypes and determine whether its components correlate with fibrosis, severity, or progesterone resistance (Feng et al., 2025; Patel et al., 2017; Marquardt et al., 2019). The proposed ability of OTUB1 to stabilise both YAP1 and estrogen receptor alpha (ERα) also warrants direct investigation because it may connect Hippo-dependent programmes with hormonal signalling (Marquardt et al., 2019; Feng et al., 2025). Such studies should distinguish epithelial, stromal, immune, and fibrotic compartments and should avoid extrapolating subtype-specific conclusions from mixed ectopic tissue.

Immune-cell and exosome-mediated regulation is another priority. In the available study, restoration of exosomal miR-196a-5p activated Hippo signalling and shifted macrophages away from an M2-like phenotype, whereas inhibition of Hippo signalling attenuated this effect (Lu et al., 2025). Future work should determine whether this mechanism is reproducible across lesion subtypes and whether other exosomal miRNAs exert opposing effects. More broadly, circRNA and miRNA networks should be evaluated using independent patient cohorts and functional models rather than inferred from pathway-enrichment analyses alone (Memczak et al., 2013; Conn et al., 2015; Zhang et al., 2018; Wang et al., 2020).

Biomarker development also requires cautious validation. Circulating YAP/TAZ-regulated factors, including miR-21-5p, and tissue YAP1 expression are candidate biomarkers rather than established diagnostic or predictive tests. Large, prospectively characterised cohorts with lesion-subtype annotation, cycle-phase information, and treatment-response data are needed to assess analytical validity, clinical validity, and added value beyond current diagnostic methods (Lin et al., 2023; Zeng et al., 2020).

## 6 Challenges and limitations

Several challenges currently limit clinical translation. Most evidence derives from *in vitro* experiments, patient-derived tissues, or animal models, and no Hippo-targeted intervention has been validated in clinical trials for endometriosis. Hippo signalling is also essential for normal tissue homeostasis, regeneration, wound healing, and stem-cell maintenance. Systemic inhibition of YAP/TAZ–TEAD activity may, therefore, produce both on-target physiological disruption and off-target toxicity. Tissue-selective delivery, exposure control, and long-term reproductive and systemic safety assessment are prerequisites for further development (Baroja et al., 2024; Paul et al., 2025).

Verteporfin was developed as a photosensitiser for photodynamic therapy and is not a selective Hippo pathway inhibitor (Michels and Schmidt-Erfurth, 2001). Its reported YAP-related effects may therefore coexist with Hippo-independent mechanisms, and systemic administration requires evaluation of phototoxicity, tissue distribution, long-term safety, and interference with physiological regeneration. HPS poses different translational challenges because it is a complex formulation with uncertain active constituents, pharmacokinetics and target specificity, and its endometriosis evidence is limited to one preclinical study. These limitations preclude presenting either approach as an available non-hormonal treatment.

Collectively, these limitations support a staged translational pathway: independent mechanistic replication, validation in subtype-resolved human models, pharmacokinetic and toxicological characterisation, tissue-selective delivery studies, and only then appropriately designed clinical investigation. Until these steps are completed, Hippo-directed strategies should be described as experimental or preclinical candidates.

## 7 Conclusion

Current management of endometriosis relies primarily on hormonal suppression, analgesia, and surgery, which can reduce symptoms or lesion burden but do not directly target the molecular programmes that sustain chronic inflammation, fibrosis, and recurrence (Dunselman et al., 2014). This limitation, together with the chronic and heterogeneous nature of the disease (Falcone and Flyckt, 2018), supports the investigation of non-hormonal mechanisms. In this context, the emerging concept of endometriosis as an inflammatory–fibrotic disorder provides a strong rationale for examining mechanotransduction and Hippo signalling (Chapron et al., 2019).

Importantly, the clinical relevance of lesion-directed interventions varies according to disease phenotype and the mechanisms underlying symptoms. For example, in superficial peritoneal endometriosis, the benefit of laparoscopic treatment for pain remains debated because pain is multifactorial and lesion-specific evidence is limited (Horne et al., 2019). Similarly, molecularly targeted approaches should be viewed as potential complements to, rather than replacements for, established management strategies.

Endometriosis subtypes—including superficial peritoneal endometriosis (SPE), ovarian endometrioma (OE), and deep endometriosis (DE)—differ substantially in tissue architecture, fibrosis, invasiveness, and hormonal responsiveness, yet direct comparisons of Hippo pathway activity across these phenotypes remain scarce (Nisolle and Donnez, 1997; Habiba et al., 2023). Subtype-resolved analyses using patient tissues and physiologically relevant models will, therefore, be essential to determine whether YAP/TAZ activation represents a common pathogenic mechanism or is preferentially associated with fibrotic and invasive disease.

Overall, current evidence supports Hippo pathway dysregulation as a plausible molecular axis integrating mechanotransduction, cell survival, fibrosis, immune regulation, and hormone resistance in endometriosis. However, the strength of evidence remains heterogeneous: while some associations are supported by patient-derived samples or endometriosis-specific experimental models, others are extrapolated from cancer and fibrotic diseases. No Hippo-targeted therapy or biomarker has yet been clinically validated in endometriosis. Thus, the Hippo pathway should presently be regarded as a compelling mechanistic and therapeutic research framework whose clinical relevance will depend on independent validation, subtype-specific investigation, and rigorous assessment of efficacy and safety.

## Glossary

ANTXR2: Anthrax toxin receptor 2
ASPP2: Apoptosis-stimulating protein of p53-2
BMI: Body mass index
circRNA: Circular RNA
circATRNL1: Circular RNA derived from ATRNL1
CTGF: Connective tissue growth factor
DE: Deep infiltrating endometriosis
ECM: Extracellular matrix
EMT: Epithelial–mesenchymal transition
ERα: Estrogen receptor alpha
ESR2: Estrogen receptor beta
ESCs: Endometrial stromal cells
EZH2: Enhancer of zeste homolog 2
FAP: Fibroblast activation protein-alpha
FDG: Fluorodeoxyglucose
HIF-1α: Hypoxia-inducible factor 1-alpha
HPS: Hu-Po-San
LATS1/2: Large tumour suppressor kinases 1 and 2
MAP4Ks: Mitogen-activated protein kinase kinase kinase kinases
METTL3: Methyltransferase-like 3
METTL14: Methyltransferase-like 14
m6A: N6-methyladenosine
miRNA: MicroRNA
MOB1A/B: MOB kinase activator 1A/B
mTOR: Mechanistic target of rapamycin
MRI: Magnetic resonance imaging
MST1/2: Mammalian STE20-like kinases 1 and 2
NCOA4: Nuclear receptor coactivator 4
NE: Normal endometrium
OE: Ovarian endometrioma
OTUB1: OTU domain-containing ubiquitin aldehyde-binding protein 1
PET-CT: Positron emission tomography–computed tomography
PGR: Progesterone receptor
ROS: Reactive oxygen species
rASRM: Revised American Society for Reproductive Medicine
SAV1: Salvador homolog 1
siRNA: Small interfering RNA
SOX18: SRY-box transcription factor 18
SPE: Superficial peritoneal endometriosis
STRIPAK: Striatin-interacting phosphatase and kinase complex
TAO: Thousand-and-one amino acid kinases
TAZ: Transcriptional co-activator with PDZ-binding motif
TEAD: TEA domain transcription factor
TIAR: Tissue injury and repair
USP33: Ubiquitin-specific peptidase 33
WWTR1: WW domain-containing transcription regulator 1
YAP1: Yes-associated protein 1
